# Dissociable Effects of Valence and Arousal in Adaptive Executive Control

**DOI:** 10.1371/journal.pone.0029287

**Published:** 2011-12-21

**Authors:** Christof Kuhbandner, Michael Zehetleitner

**Affiliations:** Department of Psychology, University of Munich, Munich, Germany; University of Granada, Spain

## Abstract

**Background:**

Based on introspectionist, semantic, and psychophysiological experimental frameworks, it has long been assumed that all affective states derive from two independent basic dimensions, valence and arousal. However, until now, no study has investigated whether valence and arousal are also dissociable at the level of affect-related changes in cognitive processing.

**Methodology/Principal Findings:**

We examined how changes in both valence (negative vs. positive) and arousal (low vs. high) influence performance in tasks requiring executive control because recent research indicates that two dissociable cognitive components are involved in the regulation of task performance: amount of current control (i.e., strength of filtering goal-irrelevant signals) and control adaptation (i.e., strength of maintaining current goals over time). Using a visual pop-out distractor task, we found that control is exclusively modulated by arousal because interference by goal-irrelevant signals was largest in high arousal states, independently of valence. By contrast, control adaptation is exclusively modulated by valence because the increase in control after trials in which goal-irrelevant signals were present was largest in negative states, independent of arousal. A Monte Carlo simulation revealed that differential effects of two experimental factors on control and control adaptation can be dissociated if there is no correlation between empirical interference and conflict-driven modulation of interference, which was the case in the present data. Consequently, the observed effects of valence and arousal on adaptive executive control are indeed dissociable.

**Conclusions/Significance:**

These findings indicate that affective influences on cognitive processes can be driven by independent effects of variations in valence and arousal, which may resolve several heterogeneous findings observed in previous studies on affect-cognition interactions.

## Introduction

Affective states can be characterized by two independent dimensions, valence and arousal [1], [2]. Valence refers to the hedonic tone of an experience ranging from negative/unpleasant to positive/pleasant, arousal refers to a sense of mobilization and ranges from low/deactivated to high/activated. All emotions can be understood as combinations of these two basic dimensions [3]. Happiness, for example, can be conceptualized as a positive emotional state involving high arousal, sadness as a negative emotional state involving low arousal. The two-dimensional model of affect has originally been derived from factor analyses of self reports of affective states and multidimensional scaling of similarity ratings of emotion-related language [1], [4]–[6], and it has been shown that the two-dimensional structure of subjective emotional experiences is mirrored in peripheral physiological reactions which are also differentially correlated along the two dimensions of valence and arousal [7]–[9]. More recently, biological correlates for the two dimensions of emotional experience have been found, indicating that valence and arousal derive from transient alterations in two independent neurophysiological systems. Valence correlates mainly with activation of the orbitofrontal cortex and is associated with the mesolimbic dopamine system, whereas arousal correlates with activation of the amygdala and is associated with the mesencephalic reticular activating system [10]–[13].

The independence of the affective dimensions of valence and arousal has been demonstrated for emotion-related changes at the levels of subjective experiences, physiological reactions, and central nervous system activations. However, it is currently unknown whether valence and arousal are also dissociable at the level of emotion-related changes in cognitive processing, which represents another central component of emotional reactions (e.g., [14]). Although it has been frequently demonstrated that emotional states are associated with changes of a wide range of cognitive processes like, e.g., perception [15]–[18], spatial or temporal attention [19]–[21], and memory [22]–[26], until now no study has investigated whether effects of valence and arousal on cognitive processing are indeed dissociable. To address the question of dissociable cognitive effects, two requirements have to be met: first, a two-by-two crossed manipulation of valence and arousal has to be performed, and second, two dependent variables have to be examined which reflect independent cognitive functions. However, the existing studies are limited on that point either in their manipulations or measurements. Most of the existing studies did not investigate the full factorial combination of negative/positive valence and low/high arousal (e.g., [16]–[18], [21], [22], [27], [28]) and second, all but one investigated only one cognitive function (i.e., one dependent variable). In particular, even that one study which investigated two dependent variables did not report dissociable effect of valence and arousal but effects of valence only [19].

The aim of the present study was to address the issue of dissociable effects of valence and arousal on cognitive processing. As mentioned above, to investigate this question, a cognitive ability has to be examined which depends on two independent cognitive processes. We choose the area of adaptive executive control because recent research suggests that the executive mechanisms involved in the adaptive regulation of task performance can be dissociated in two components: a control process which makes sure that only task-relevant signals are selected, and a conflict-monitoring process which determines how much control is exerted and when control is withdrawn [29], [30]. Empirically, the strength of control is reflected by the amount of interference by task-irrelevant stimuli, and the strength of conflict-driven control adaptation by the modulation of interference after trials in which conflicting stimuli were present (we use the terms ‘control’ and ‘control adaptation’ throughout the article to refer to the theoretical cognitive constructs, and the terms ‘interference’ and ‘modulation of interference’ to refer to the empirical operationalizations of these theoretical constructs). On the basis of prior theorizing, we expected that arousal might influence control whereas valence might influence control adaptation. It is often assumed that one core component of high arousal is a strong responsiveness to sensory stimuli [31]. Accordingly, high levels of arousal might impede the filtering of task-irrelevant signals by cognitive control. Valence has been assumed to modulate the balance between goal maintenance and flexibility [32]. Accordingly, higher levels of pleasure might impede conflict-based control adaptation to promote cognitive flexibility.

In order to determine whether valence and arousal are dissociable in their effects on control and control adaptation, an empirical and a conceptual step are necessary: Empirically, it has to be shown that valence and arousal differentially influence the two dependent variables reflecting variations in control and control adaptation, interference and conflict-driven modulation of interference. However, there is no one-to-one mapping of the cognitive functions of control and control adaptation to the empirical variables interference and conflict-driven interference modulation. Therefore, an additional conceptual step is to identify the empirical conditions which have to be satisfied to conclude that differential effects on empirical interference and conflict-driven interference modulation can indeed be attributed to dissociable cognitive functions, rather than to a combination of cognitive functions.

Empirically, to examine the effects of valence and arousal on interference and conflict-driven interference modulation, we first induced four different affective states derived by crossing the two dimensions of valence and arousal: happiness (positive valence, high arousal), anxiety (negative valence, high arousal), calmness (positive valence, low arousal), and sadness (negative valence, low arousal). Directly after affect induction, participants performed a visual pop-out distractor task in which substantial interference effects and conflict adaptation effects typically occur [33], [34]. Observers were instructed to search for a pop-out target defined in a specific dimension (a tilted among vertical gray bars). In half of the trials, the search array contained an additional task-irrelevant pop-out distractor defined in a different dimension (a white among gray vertical bars). In trials in which the pop-out distractor is present, search performance is typically slowed, which reflects interference by salient, but task-irrelevant, stimuli [35]. After trials following such a distracting event, interference is typically reduced, which indicates that observers are able to adaptively modulate the amount of current control by conflict-driven control adaptation [30], [33], [34]. Neurophysiological evidence suggests that interference and adaptive modulation of interference reflect independent cognitive functions because different brain areas are involved: Pre-trial activity in the medial frontal cortex is a good predictor for the size of search interference on a trial-by-trial basis [36], whereas the anterior cingulate cortex (ACC) is related to adaptive control [30]. However, the independence of interference by salient distractors and its adaptive modulation has not yet been demonstrated.

Conceptually, to establish the empirical markers which allow to conclude that differential effects of valence and arousal on interference and conflict-driven modulation of interference reflect dissociable effects on the cognitive functions of control and control adaptation, we explored the dynamics of a prominent formal model of executive control [30]. According to this model, the amount of current cognitive control is dependent on (i) the base level of control, (ii) the amount of conflict in previous trials, and (iii) the strength of control adaptation (for a detailed description of the model, see below). Critically, based on the literature, the conditions are unknown allowing it to distinguish whether changes in the mean levels of control and conflict-driven modulation of control originate from the change of one model parameter or a combination of model parameters. Accordingly, we first explored the control model’s system dynamics using a Monte Carlo simulation in order to establish the conditions allowing to attribute changes in mean control and mean conflict-driven modulation of control directly to model parameters. Then, we examined whether the conditions for dissociable effects on control and control adaptation are satisfied for the effects of valence and arousal on adaptive executive control.

## Results

### Affect Induction

Participants reported the expected differences in valence and arousal after affect induction. Valence ratings were higher in the positive-affect groups (happiness: *M*  =  6.69, *SE*  =  0.34; calmness: *M*  =  6.96, *SE*  =  0.32) compared to the negative-affect groups (anxiety: *M*  =  4.50, *SE*  =  0.45; sadness: *M*  =  4.24, *SE*  =  0.47), *F*(1, 98)  =  39.0, *P* < 0.001, η_p_^2^  =  .29. Arousal ratings were higher in the high-arousal groups (happiness: *M*  =  6.00, *SE*  =  0.36; anxiety: *M*  =  5.54, *SE*  =  0.45) compared to the low-arousal groups (calmness: *M*  =  3.64, *SE*  =  0.43; sadness: *M*  =  3.76, *SE*  =  0.33), *F*(1, 98)  =  28.6, *P* < 0.001, η_p_^2^ =  .23. Within the positive-affect and negative-affect groups, valence ratings did not differ, *F*s < 1, and within the low-arousal and high-arousal groups, arousal ratings did not differ, *F*s < 1.

### Pop-out Distractor Task

#### Interference

**Figure 1:**
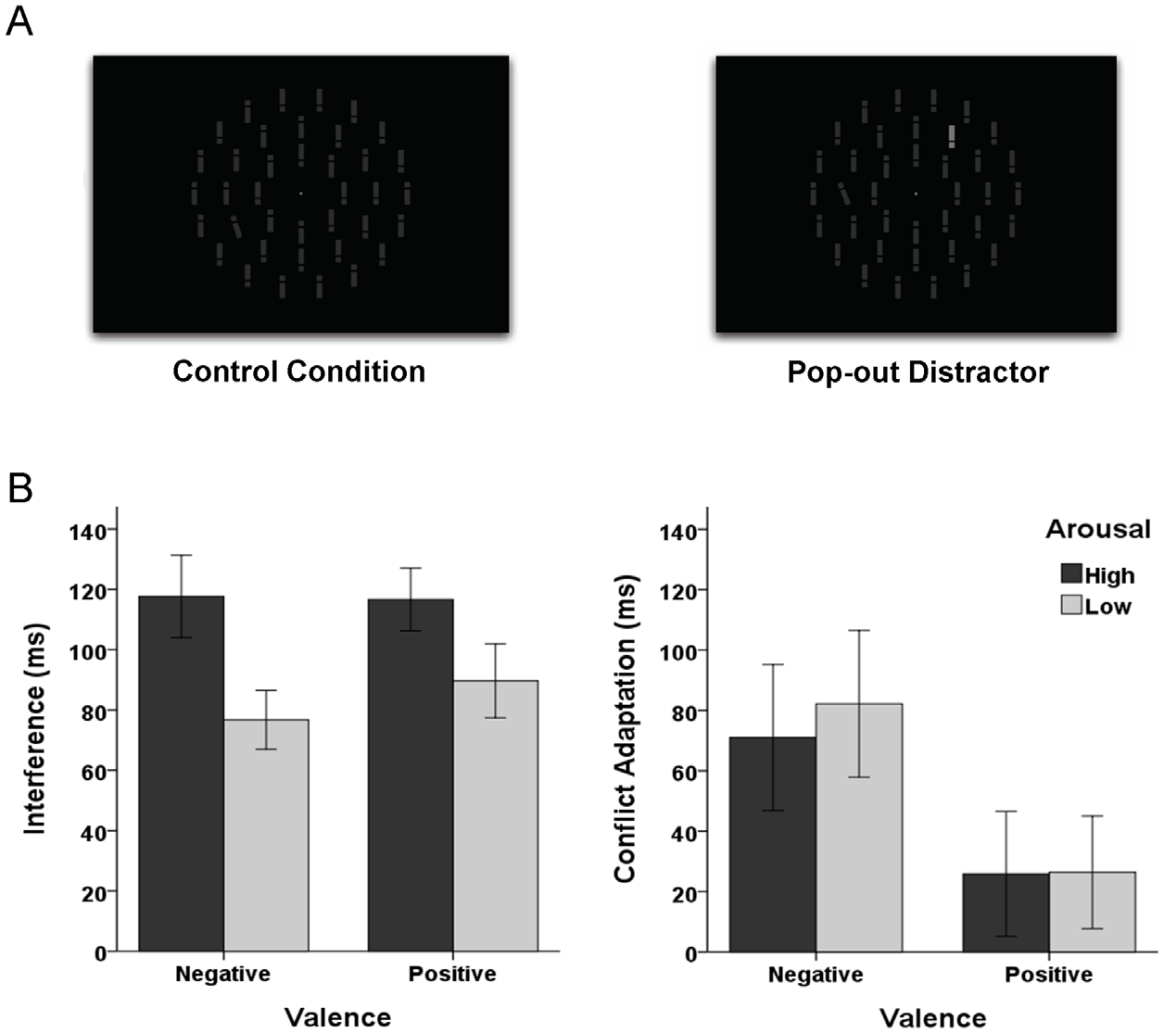
Experimental materials and results. (A) Examples of search displays in the control condition (left panel) and the pop-out distractor condition (right panel). Participants were instructed to search for the tilted pop-out target and to ignore an occasionally occurring luminance pop-out distractor. (B) Left panel: Mean interference effects (reaction time on pop-out distractor trials minus reaction time on control trials) as a function of valence state (negative, positive) and arousal state (high, low). Right panel: Mean conflict-adaptation effects (interference on trials following pop-out distractor trials minus interference on trials following control trials) as a function of valence state (negative, positive) and arousal state (high, low). Error bars indicate standard errors.

Outlier trials (Reaction Times > 1400 ms; 3.9%), the first trial of each experimental block (1.6%), and errors (2.2%) were excluded from further analyses. To determined interference effects, for each participant, we first calculated mean reaction times (RTs) for distractor-present trials (RT_dis_) and mean RTs for distractor-absent trials (RT_no-dis_). Interference was then calculated by subtracting the two mean RTs: RT_dis_–RT_no-dis_. To analyze interference as a function of induced affect, interference effects were subject to an ANOVA with the between-participant factors valence and arousal, and the within-participant factor block (first vs. second block). There was a significant main effect of block, *F*(1, 96)  =  39.5, *P* < 0.001, η_p_^2^  =  .29. Interference strongly decreased across the two experimental blocks (from 100 to 51 ms), replicating previous findings that training substantially reduces the size of attentional interference [34]. This main effect was qualified by a significant block x arousal interaction, *F*(1, 96)  =  8.0, *P*  =  0.006, η_p_^2^  =  .08. Simple main effect analyses showed that interference effects were stronger in high arousal states (*M*  =  117 ms ) than in low arousal states (*M*  =  83 ms) in the first block (see Fig. 1b, left panel), *F*(1, 96)  =  8.6, *P*  =  0.004, η_p_^2^  =  .08, but not in the second block, *F* < 1, indicating that high arousal initially increased interference, but did not impair the down-regulation of interference over training. The effect of arousal was not accompanied by an effect of valence or by a valence x arousal interaction, *F*s < 1, indicating that the amount of interference was only influenced by arousal, but not by valence.

#### Conflict-driven Modulation of Interference

To determine conflict-driven modulation of interference, we compared interference effects following distractor-present trials (I_dis_) with interference effects following distractor-absent trials (I_no-dis_). Conflict-driven modulation of interference was calculated by subtracting the two measures of interference: I_no-dis_–I_dis_. An ANOVA with the within-participant factor block revealed that a reduction in interference after conflicting trials was only found in the first experimental block, but not in the second block (51 ms versus −3 ms), *F*(1, 99)  =  11.8, *P* < 0.001, η_p_^2^ =  .11, replicating previous findings that conflict-driven adaptation mechanisms are only at work as long as the suppression of distracting stimuli is not fully established by training [34]. Analyzing conflict-adaptation effects as a function of induced affect only for the first block revealed a main effect of valence, *F*(1, 96)  =  5.2, *P*  =  0.024, η_p_^2^  =  .05 (see Fig. 1b, right panel). Conflict-driven interference reductions were larger in negative states (*M*  =  76 ms) than in positive states (*M*  =  26 ms). This effect of valence was not accompanied by an effect of arousal or by a valence x arousal interaction, *F*s < 1, indicating that conflict-driven modulation of interference was only influenced by valence, but not by arousal.

#### Error Rates

We also examined whether induced affect influenced error rates. Overall, both affective effects on interference (all *P*s > .09) and conflict-driven modulation of interference (all *P*s > .24) for error rates were not modulated by affect condition.

#### Practice Task

To analyze whether the four affect induction groups might have differed already before affect induction, RTs in the practice task before affect induction were subject to an analysis of variance (ANOVA) with the between-subjects factors valence (positive vs. negative) and arousal (low vs. high). Mean RT was 806 ms and was unaffected by valence and arousal (all *F*s < 1.5, all *P*s > 0.21). The same ANOVA of error rates also revealed no significant effects (mean error rate 2.7%, all *F*s < 1.17, all *P*s > 0.28). That is, there were no significant differences between participants subsequently assigned to the different affect induction groups.

### Dissociability of Control and Control Adaptation

#### Monte Carlo Simulation

To establish the empirical conditions of when differential effects on interference and on conflict-driven modulation of interference can be attributed to dissociable cognitive functions, we explored Botvinick et al’s [29] model of executive control using a Monte Carlo simulation. We simulated an interference paradigm in which a conflict was present in 50% of all trials. Using the difference equation for conflict of [29]

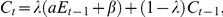
 where *C_t_* reflects control at time t as being dependent of control at the last point in time, *C_t_*_−1_, and a measure of conflict at the last point in time, *E_t_*_−1_. *λ* weights the contribution of previous control (stability) and previous conflict (flexibility) and thus reflects control adaptation. *β* is the amount of control which is added at each point in time and thus reflects the base-level of control. Parameter *α* in contrast is not related directly to control, but modulates conflict, *E*. That is, the same physical amount of conflict in the environment can be amplified or dampened, depending on *α*. In difference to the original formulation of the model, we exchanged the roles of *λ* and 1 – *λ* in order to allow *λ* to be interpreted semantically as control adaptation rather than cognitive stability/rigidity as would be necessary in the original formulation. This alteration of course leaves the model’s dynamics unchanged. In order to explore the dynamics of executive control, we simulated an interference paradigm for ca. 8000 parameter combinations of *λ*, *β*, and *α*. Each parameter was varied between 0 and 1 in 10 steps, except for *λ*, which was varied between 0.05 and 0.95. For each set of parameters, control started with 0 and a random sequence of conflict and no conflict trials was presented to the system. For conflict trials, the conflict measure *E* was set to 1, and for no-conflict trials, *E* was set to 0. The mean level of control was calculated as the average control over all 1000 trials. Mean control adaptation was calculated as the difference in mean control between trials following no- conflict trials and trials following conflict trials. Both measures relate to empirical measures in interference paradigms. RT interference is inversely proportional to mean control (the higher mean control, the smaller RT interference), mean control adaptation reflects the reduction in RT interference in trials following conflict trials compared to trials following no-conflict trials.

**Figure 2:**
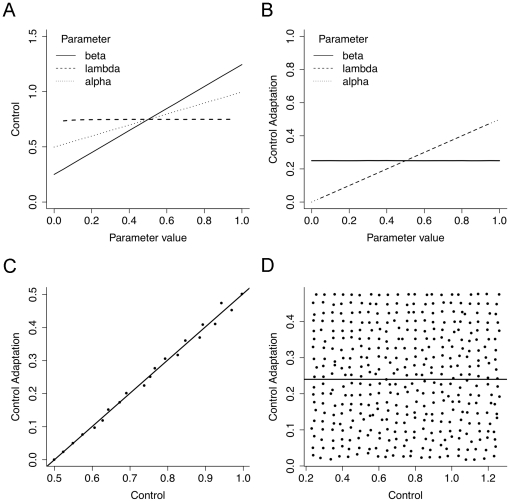
Results of the Monte Carlo simulation of Botvinick et al’s (30) model of control. (A) Effect of the parameters *β*, *λ*, and *α* on mean control. (B) Effect of *β*, *λ*, and *α* on mean control adaptation. (C) Correlation between mean control and mean control adaptation for fixed *β*, *λ* with variable *α*. (D) Correlation between mean control and mean control adaptation for a fixed level of *α*.

Figure 2 presents how the parameters *λ*, *β*, and *α* affect mean control and mean control adaptation in the Monte Carlo simulation. Mean control is affected only by parameters *β* and *α*, but not by parameter *λ* (see Fig. 2a). On the other hand, mean control adaptation is affected by *λ* and *α*, but not by parameter *β* (see Fig. 2b). Put semantically, the higher the base-level of control, *β*, the higher the mean level of control becomes, whereas mean control adaptation remains unaffected. By contrast, the higher the level of control adaptation, *λ*, the stronger mean control adaptation becomes, whereas the mean level of control remains unaffected. Finally, a stronger emphasis of conflict (i.e., an increase in parameter *α*) increases both the mean levels of control as well as conflict adaptation. A pure variation of *α* leads to a near perfect correlation between mean control and mean conflict adaptation. For each combination of *λ* and *β*, the correlation coefficient is greater than .95 (except for the highest levels of *λ*, where the correlation coefficient is always greater than .85; see Fig. 2c). However, the simulation revealed that if *α* is fixed and only *λ* and *β* are allowed to vary, there is no correlation between mean control and mean conflict adaptation (see Fig. 2d), indicating that *λ* and *β* can indeed be dissociated when this condition is satisfied. This holds true for every level of *α*, the correlation coefficients are always below .01. In summary, it can be concluded that two experimental factors have dissociable effects on adaptive executive control if three conditions are satisfied: (i) one factor affects interference but not conflict-driven interference modulation, (ii) the other factor affects conflict-driven interference modulation but not interference, and (iii) there is no correlation between interference and conflict-driven interference modulation.

### Dissociability of Valence and Arousal Effects

To examine whether the empirical criterion for dissociable effects of two experimental factors on control and control adaptation are satisfied in the present study, we correlated interference effects with conflict-driven interference modulation effects in the first experimental block. The analysis revealed that both measures of executive control were indeed uncorrelated, *r*  =  0.04, *P*  =  0.647, indicating that the effects of arousal and valence on control and control adaptation are indeed dissociable.

## Discussion

It has long been claimed that affective experiences derive from two independent basic dimensions, valence and arousal. Indeed, based on introspectionist [5], semantic [4], and psychophysiological [9] experimental frameworks, previous research has shown that valence and arousal are indeed dissociable at the levels of emotion-related changes in subjective experiences, physiological reactions, and central nervous system activations. The results of the present study demonstrate that valence and arousal can also be dissociated at the level of emotion-related changes in cognitive processing. Using a visual pop-out distractor task, we found that valence and arousal differentially influenced the two basic requirements of adaptive executive control: Control (i.e., strength of filtering goal-irrelevant signals) and control adaptation (i.e., strength of maintaining current goals over time). Involuntary attentional capture by goal-irrelevant pop-out distractors was largest in high arousal states, independently of valence, indicating that arousal, but not valence, modulates control. By contrast, the increase in control after trials in which goal-irrelevant stimuli were present was largest in negative states, independently of arousal, indicating that valence, but not arousal, modulates control adaptation. A Monte Carlo simulation revealed that these observed effects of valence and arousal on adaptive executive control are indeed dissociable. The simulation revealed that effects of two experimental factors on control and control adaptation are dissociable if there is no correlation between empirical interference and the conflict-driven modulation of interference, which was indeed the case in our study.

The finding that distraction by goal-irrelevant pop-out distractors was highest in happy states and lowest in sad states corroberates other findings demonstrating that negative affect compared to positive affect broadens selective processing in different cognitive domains like, e.g., iconic memory [16], spatial attention [37], semantic memory [38], or action representation [39], and is consistent with the broaden-and-build theory [40] positing that one of the primary function of positive affect is to broaden a person’s thought-action repertoire and to build personal resources by making people more open-minded and more sensible for opportunities in the environment. Our results suggests, however, that such affective influences on the breadth of selective processing can be driven by independent effects of variations in the two underlying dimensions of valence and arousal. Accordingly, depending on the demands a task makes on executive control, selective processing can be broadened either due to a weak level of attentional control, due to a weak level of control adaptation, or both.

Indeed, this might also account for the rather heterogeneous findings observed in the few studies investigating the full factorial combination between positive/negative valence and high/low arousal. For instance, studies using false memory tasks or semantic generation tasks suggest that the breadth of processing is modulated by arousal rather than by valence, because false memories or unusual word-associations are more frequent in high than in low arousal states, irrespective of their valence [25], [38]. By contrast, studies using attentional blink tasks suggest that arousal and valence interact in their effects on the breadth of processing because sadness seems to broaden processing (i.e., reduce the attentional blink), whereas anxiety seems to narrow processing (i.e., enhance the attentional blink), with calm and happy states in-between [20]. However, this mixed pattern of results is not surprising as the requirements for executive control are rather different between both type of tasks. The occurrence of false memories and the production of unusual associates should only vary with the level of control because both phenomenons depend on the activation of irrelevant signals which is not experienced as a conflict [41]. Accordingly, performance should be influenced by arousal rather than by valence. By contrast, the size of the attentional blink depends both on the level of control and on the level of control adaptation. Attentional blink effects are stronger if there is an overinvestment of general attentional resources in the task because this increases interference by task-irrelevant items of the rapidly presented sequence of stimuli [42]. Accordingly, attentional blink should be modulated by valence because the strength of maintaining current attentional goals over time varies depending on valence. However, the amount of interference when paying a high amount of attention to the task should additionally vary with arousal because the base level of control should depend on arousal.

Our results indicate that interference by task-irrelevant stimuli can be large both in high-arousal positive states and in high-arousal negative states. At a first glance, this might seem surprising as it has been hypothesized that high arousal in negative states is associated with a constriction of attentional focus, leading to a focusing on task-relevant cues and an exclusion of task-irrelevant cues (i.e., cue-utilization hypothesis; [43]). A prominent example thereof is the so-called “weapon focus” which refers to the phenomenon that attention in high arousing situations like an attack is narrowed to central details (i.e., the weapon) at the expense of peripheral details [44]. However, more recently, it has been suggested that such attentional narrowing is not inevitably produced by high-arousal negative states. Instead, such effects seem only to occur if central stimuli are highly salient [45]. In line with these findings, our study demonstrates that high arousal in negative states is not inevitably associated with a narrowing of attentional focus. By contrast, at least if task-irrelevant stimuli are salient, then high arousal seems even to broaden attention. In particular, this would suggest that the critical dimension influencing attentional selection of cues in highly arousing situation is not goal-driven task relevance, but rather stimulus-driven salience of a stimulus. Indeed, determining the role of salience in emotional effects on attention might be an interesting avenue for future research.

The possible critical role of salience of task-irrelevant stimuli might also explain why the only one existing study examining the influence of valence and arousal on control and control adaptation did not report dissociable effect of valence and arousal [19]. Using a flanker task [46], it was found that observers in negative states showed stronger control adaptation after a conflict than observers in positive states, which is consistent with the valence effect in the present study. However, different from the present study, the base level of control was not influenced by arousal. The reason for the failure to find effects of arousal on control might be that van Steenbergen and colleagues used distractor stimuli with low salience (i.e., different color words as targets and distractors). Indeed, compared to the mean interference effects in the present study (100 ms), mean interference effects in that study were rather small (34 ms). Thus, one important prerequisite for the occurrence of arousal effects on distraction by task-irrelevant stimuli seems to be their high salience.

The finding of dissociable effects of valence and arousal in adaptive executive control is well in line with findings demonstrating that valence and arousal are associated with different neurotransmitter systems which are known to differentially modulate control and control adaptation. Valence is associated with changes in the activity of the dopamine system (e.g., [47]), which is assumed to play an important role in the modulation of cognitive flexibility [48]. Arousal is associated with changes in the activity of the norepinephrine system (e.g., [49]), which is assumed to play an important role in alerting the cortex to attend to salient sensory stimuli [50]. Accordingly, it might be that the effects of valence and arousal on control and control adaptation are mediated by effects of valence and arousal on dopamine and norepinephrine release. Thus, one interesting topic for future research is to determine the neurophysiological background of how valence and arousal act on adaptive executive control.

The results of the present study replicate previous findings demonstrating that training can substantially reduce the size of attentional interference, and that conflict-driven adaptation mechanisms are only at work as long as the suppression of distracting pop-out stimuli is not fully established [34]. Interference substantially decreased from the first to the second experimental block, and a decrease in interference after distracting trials was only observed in the first experimental block. In particular, our results suggest that although high arousal initially increases interference, it does not impair the down-regulation of interference over training, because effects of arousal on interference were found only in the first experimental block. However, one might argue that the fact that no effects of arousal were found in the second block might merely reflect the fading of affect induction effects. Indeed, it is often found that induced affect can fade relatively quickly, lasting typically less than ten minutes (e.g., [51], [52]). However, as our experimental task was very short and lasted less than two minutes (first experimental Block: *M*  =  0.96 min, second experimental block: *M*  =  0.94 min; no break between blocks), it seems unlikely that the induced affective state substantially faded during the experimental task.

The results of our study support dimensional models of affect positing that all affective states derive from the two independent basic dimensions of valence and arousal. Dimensional models of affect are consistent with many recent findings from behavioral, cognitive neuroscience, neuroimaging, and developmental studies of affect (e.g., [53]), and the current study adds to this voluminous body of research by demonstrating that valence and arousal are also dissociable in their effects on cognitive functions. However, there is still a debate whether all affective experiences arise from the two underlying dimensions of valence and arousal, or whether there is a core set of basic emotions which are distinct and independent from each other (e.g., [54], [55]). Indeed, one could argue that the effects reported for the four different induced affective states represent specific effects of the respective emotions, although this would be the less parsimonious explanation. One interesting question for further research would be to examine whether affective states characterized by similar valence and arousal values like, e.g., anxiety and anger, have similar effects on executive control functions, because this would further support dimensional models of affect.

In conclusion, the present study demonstrates that the basic dimensions of affect, valence and arousal, can also be dissociated in their effects on cognitive functions. In particular, our results indicate that affect-induced modulations of performance in tasks requiring executive control can be can be driven by independent effects of variations in the two underlying dimensions of valence and arousal, which underlines the importance of investigating the full factorial combination of valence and arousal when examining affective influences on cognitive processing.

## Materials and Methods

### Participants and Design

104 undergraduate students were randomly assigned to one of four affect conditions (happiness, anxiety, calmness, sadness). All reported normal or corrected-to-normal vision and received either course credit or monetary reward (8 Euro) for participation. Data from four participants were excluded from analyses because their error rate deviated more than two standard deviations from the mean error rate, resulting in a final sample of 100 participants (67 female, mean age  =  24.81 years, *SD*  =  5.48). This research was approved by the ethic's committee of the University of Munich (LMU), and all participants provided informed written consent.

### Affect Induction

A standard affect-induction procedure was used that combines music with imagination [56]. Participants were instructed to recall in detail an autobiographical event while listening to music. The music and the autobiographical event were either happy, frightening, calm, or sad (details of instruction and musical selections in [20]). Subsequent to affect induction, the success of induction was measured using the affect grid [57], which assesses current affect on the dimensions of valence (1  =  extremely negative, 9  =  extremely positive) and arousal (1  =  low arousal, 9  =  high arousal).

### Pop-out Distractor Task

Stimuli were presented on a 19 inch cathode ray monitor. Stimulus presentation and response recording was controlled by software purpose written in C++. In each trial, observers had to report the identity of an orientation feature singleton (“pop-out” target). In half of all trials, a task-irrelevant luminance pop-out distractor could also be present and had to be ignored by the observer (see Fig. 1a). Non-targets were upright rectangle forms (bars) which had a gap either at the top or at the bottom of the bar (roughly resembling the letter ‘i’ or an exclamation mark ‘!’). Stimuli were 0.25° of visual angle wide and 1.25° high with a luminance of 5 cd/m2. The orientation target differed from non-targets in tilt and was oblique 15° either to the left or to the right of vertical. In a pilot experiment set size was manipulated to ensure that the 15° tilt target was efficient in the sense that increasing the number of distractors to be searched did not slow search times [58]. The irrelevant target differed from non-targets in luminance (35 cd/m2). Each search display consisted of a homogeneous arrangement of thirty-six bars placed on three (invisible) concentric rings about a fixation point. The first, second, and third ring had 6, 12, and 18 elements with a distance of 1.75°, 3.25°, and 4.76° from the fixation point. Targets were placed randomly (uniform distribution) on one of the 12 locations of the second ring, and the irrelevant singleton, if present, on one of the 11 remaining locations of the same ring. Observers had to indicate by clicking a mouse button, whether the orientation target was of the shape ‘i’ or ‘!’ as fast and as accurately as possible. Each trial started with a fixation point (diameter 0.2° visual angle) for a mean presentation time of 1000 ms (uniformly distributed between 800–1200 ms). The search display was present until the observers responded.

Participants first practiced the search task for four blocks of 32 trials each during which only targets were presented and additional irrelevant stimuli never appeared. Afterwards, affect induction took place, followed by two experimental blocks of 32 trials each.
